# Benzalkonium Chloride Significantly Improves Environmental DNA Detection from Schistosomiasis Snail Vectors in Freshwater Samples

**DOI:** 10.3390/tropicalmed10080201

**Published:** 2025-07-22

**Authors:** Raquel Sánchez-Marqués, Pablo Fernando Cuervo, Alejandra De Elías-Escribano, Alberto Martínez-Ortí, Patricio Artigas, Maria Cecilia Fantozzi, Santiago Mas-Coma, Maria Dolores Bargues

**Affiliations:** 1 Departamento de Parasitología, Facultad de Farmacia, Universidad de Valencia, Av. Vicente Andrés Estellés s/n, Burjassot, 46100 Valencia, Spain; raquelsanmarques22@gmail.com (R.S.-M.); alejandra.elias@uv.es (A.D.E.-E.); alberto.martinez@uv.es (A.M.-O.); patricio.artigas@uv.es (P.A.); maria.cecilia.fanozzi@uv.es (M.C.F.); s.mas.coma@uv.es (S.M.-C.); 2 CIBER de Enfermedades Infecciosas, Instituto de Salud Carlos III, C/ Monforte de Lemos 3-5. Pabellón 11. Planta 0, 28029 Madrid, Spain; 3 Departamento de Zoología, Facultad de Biología, Universidad de Valencia y MVHN-i\Biotaxa, C/Doctor Moliner 50, Burjassot, 46100 Valencia, Spain

**Keywords:** environmental DNA, freshwater samples, benzalkonium chloride, qPCR, experimental validation, *Bulinus truncatus* survey and surveillance

## Abstract

Urogenital schistosomiasis, caused by *Schistosoma haematobium* and transmitted by *Bulinus* snails, affects approximately 190 million individuals globally and remains a major public health concern. Effective surveillance of snail vectors is critical for disease control, but traditional identification methods are time-intensive and require specialized expertise. Environmental DNA (eDNA) detection using qPCR has emerged as a promising alternative for large-scale vector surveillance. To prevent eDNA degradation, benzalkonium chloride (BAC) has been proposed as a preservative, though its efficacy with schistosomiasis snail vectors has not been evaluated. This study tested the impact of BAC (0.01%) on the stability of *Bulinus truncatus* eDNA under simulated field conditions. Water samples from aquaria with varying snail densities (0.5–30 snails/L) were stored up to 42 days with BAC. eDNA detection via qPCR and multivariable linear mixed regression analysis revealed that BAC enhanced eDNA stability. eDNA was detectable up to 42 days in samples with ≥1 snail/L and up to 35 days at 0.5 snails/L. Additionally, a positive correlation between snail density and eDNA concentration was observed. These findings support the development of robust eDNA sampling protocols for field surveillance, enabling effective monitoring in remote areas and potentially distinguishing between low- and high-risk schistosomiasis transmission zones.

## 1. Introduction

Urogenital schistosomiasis is a snail-borne disease caused by the parasite *Schistosoma haematobium* (Trematoda, Schistosomatidae). It is a NTD affecting around 190 million people, thus being one of the most prevalent parasitic diseases in the world [1]. The life cycle of *S. haematobium* includes freshwater snails as intermediate hosts (functionally considered as vectors), among which *Bulinus truncatus* (Gastropoda, Bulinidae) is the main and most widespread in all endemic areas [2]. Therefore, the sole presence of this snail species in a water body, with the concomitant presence of definitive infected hosts, represent a risk for the nearby population due to its role in the transmission of schistosomiasis.

Although the distribution of this vector has historically been limited to tropical and subtropical areas, climate change and globalization appear to be contributing to its expansion to other regions [3,4]. Due to the socio-economic effects of schistosomiasis infections, an accurate disease surveillance and prevention plan is key to its control and human infection elimination, as stated by the World Health Organization (WHO) in the road map for NTD 2021–2030 [5]. Determining the risk areas of endemic countries is the first step to designate priority areas for mass drug administration (MDA) and water and sanitation actions. Traditionally, it has been performed by expert malacologists who searched for snails that could transmit the parasite in suspicious water bodies. However, these monitoring methods present several limitations: (i) they are highly time-consuming, (ii) there is a limited number of trained malacologists available to perform such work, (iii) the physical and ecological characteristics of the water bodies can hinder accurate detection of vector snails, and (iv) the natural cercarial shedding test may yield false negatives, as only a small proportion of snails are typically infected at any given time, potentially leading to an underestimation of human infection risk [6,7,8]. Given the importance of snail vector surveillance, extensive efforts have been focused on developing new methods and techniques to overcome these limitations.

To enhance the effectiveness of control and elimination strategies targeting parasites of medical and veterinary significance, there is a growing demand for more rigorous and systematic research into environmental transmission pathways [9]. This entails the necessity of detecting and monitoring the free-living, infective stages of parasites, along with their environmental vectors. The application of eDNA methods in the field of parasitology and disease surveillance has only begun to be explored [10,11].

Detection of eDNA of schistosomiasis vectors in fresh water using qPCR has emerged as one of the most promising tools for epidemiological surveillance [11], leaving behind the exhausting process of conventional malacological surveillance through visual examination of water bodies. In fact, a recent study commissioned by the WHO, with the aim of determining the best way to diagnose schistosome infections in snails and the ability of a water body to be a site of transmission, ranked eDNA analysis as one of the three most encouraging techniques [12]. The value of the eDNA technique as a surveillance and xenomonitoring tool to determine the potential risk of transmission in water bodies is due to (i) the ease of collecting water samples, (ii) the elimination of errors in human visual examination, (iii) the seasonality of the snail population dynamics, and (iv) the specificity and sensitivity of qPCR compared to conventional PCR. The necessity for a sensitive technique is paramount when a small population is present, as the typical concentration of eDNA is considerably lower than that of genomic DNA within cells. This is the key to ensuring successful detection [12].

For many parasitic diseases, global changes lead to a spatial redistribution of the vectors that transmit the diseases and may also lead to disease emergence [13,14,15]. This is particularly crucial in light of the emergence of schistosomiasis in non-endemic countries [16,17,18], such as France and Spain, where there is a pressing need for rapid and accurate surveillance [8,19,20,21,22,23,24].

On-site field extraction and qPCR technologies are particularly valuable in monitoring situations that are time-sensitive or involve sample collection in remote areas where sample degradation could pose significant challenges [25]. Although field extraction and qPCR technologies exist to facilitate on-site analysis [26], the need to assess the post-collection stability of samples and to standardize the technique has been emphasized [27], particularly given that endemic areas are often situated far from reference laboratories. These regions commonly face logistical and climatic challenges that hinder the maintenance of the cold chain, which is essential for preserving the integrity of genetic material [12].

Recent evidence indicates that BAC has the potential to inhibit the degradation of fish eDNA from freshwater environments by modulating the activity of DNase, thereby mitigating its degrading effect on DNA. However, the efficacy of this approach has only been tested in the context of ecological surveillance of different types of fish [28]. It provides a promising solution to the aforementioned limitation and could be applied to the surveillance of *Bulinus* spp. through eDNA detection. However, previous studies on schistosomiasis and its snail vectors have analyzed eDNA after immediately filtering the sampled water in the field [29,30,31], but the convenience of BAC as preservative with this purpose has not been tested before.

In this study, we aim to develop a water-sample collection protocol for the long-term detection of snail vector eDNA by extrapolating findings from laboratory experiments mirroring field conditions, aiming to overcome the eDNA instability encountered during field work. We primarily investigate the effect of adding the DNA preservative BAC (0.01%) on two main aspects: (i) assessing its influence on the decay rate of eDNA in water samples and (ii) examining how it affects the sensitivity of the eDNA detection technique. Additionally, we assess the correlation between snail density and the detection of eDNA, to elucidate how the density of these organisms influences eDNA detection in the environment.

## 2. Materials and Methods

### 2.1. Experiment Design

To test the influence of the addition of an eDNA preservative (BAC) on the decay rate and sensitivity of eDNA, and of the effect of snail density on eDNA detection, laboratory set-ups were performed using *B. truncatus* snails from Almería, Spain, born and reared in laboratory conditions and measuring between 3.5 and 6 mm (Supplementary Figure S1).

The laboratory experiment was designed using 12 aquaria separated into four experimental groups: (i) three replicated aquaria containing one snail in 2 L of mineral water (density of 0.5 snails/L), (ii) three replicated aquaria with one snail in 1 L of mineral water (density of one snail/L), (iii) three replicated aquaria with 10 snails in 1 L of mineral water (density of 10 snails/L), and (iv) three replicated aquaria with 30 snails in 1 L of mineral water (density 30 of snail/L). The snail densities were selected to account for a range of variability in natural environments, considering a minimum density of 0.5 snails/L to be a fairly low density given that snails can always be found in community.

**Figure 1:**
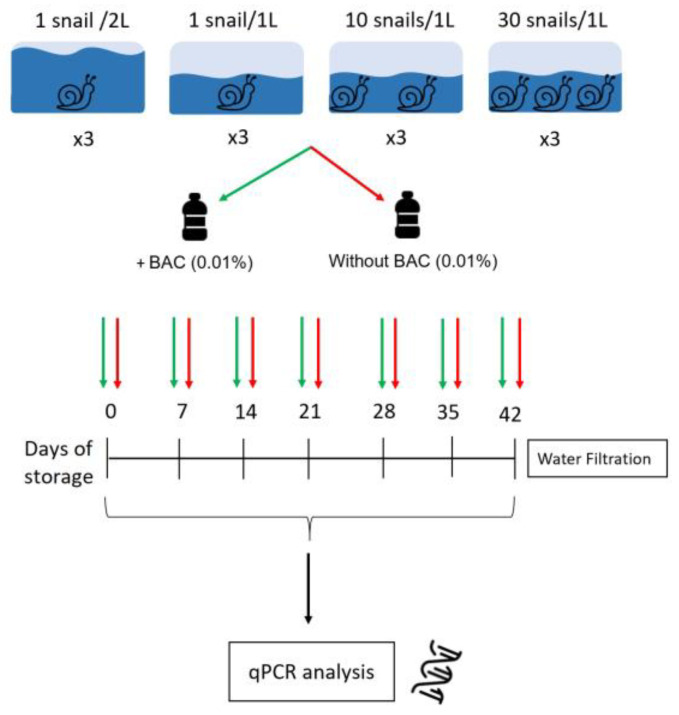
Overview of the laboratory experiment. Experiment set-up and following procedures are shown. The four snail population groups are indicated above, and their three respective replicate aquaria are indicated as “x3”. All the waters were collected in glass bottles, and BAC (0.01%) was added to the treatment group. The bottles were filtered at different times (every seven days) during a time period of 42 days. The resulting membranes were analyzed by qPCR.

All of them were kept in a climatic chamber with 24 h oxygenation, 12 h/12 h light/night cycles, and an average temperature of 20.8 °C (range of 20.1–21.7 °C). Each aquarium was maintained in these conditions for seven days, with a constant snail density, before collecting the water sample, considering the seventh day as the “day of collection” (day 0). In addition, aquaria without snails but maintained under the same conditions as the experimental aquaria were added as a negative control. A one-liter water sample was collected from each aquarium, following previous recommendations [31]. As the required number of snails was not available, the experimental procedure was conducted twice (with and without the addition of BAC), each one being carried out seven days apart. In the first experimental procedure, 1 mL of BAC with a 10% initial concentration (50 gr. of BAC in 500 mL of water, according to the manufacturer’s instructions (Sigma-Aldrich, Darmstadt, Germany)) was added to the collected water samples, for a final solution concentration of 0.01%. Those samples were considered as the “treatment group”. In the second experimental procedure, samples were collected without BAC and were referred to as the “control group”. The experiments and analytical procedures were designed to allow inter-comparison, and the snails were monitored to guarantee the same size in both groups (Figure 1).

To assess the impact of post-collection time on both groups, the water from all density aquaria was collected, whether treated or not, and stored for 0, 7, 14, 21, 28, 35, or 42 days at a medium temperature of 20 °C and under daily light conditions. After the specified time period, the entire water sample was filtered (Figure 1), and pH and temperature were measured prior to filtration.

### 2.2. Water Filtration

The filtration of the water samples was performed using a 1 L glass vacuum filter flask with side arm and a fitted glass funnel to prevent air leaks. This procedure was facilitated by applying a vacuum of −200 hPa to ease the water flow through a 47 mm diameter filtration membrane (GF/F, 0.7 μm; Whatman, Maidstone, UK). All the materials used for the process were carefully washed and sterilized with bleach (5%) between each filtration to avoid contamination. Once all the filtrations were carried out, the eDNA was fixed to the membranes by adding 70% ethanol. The membranes were individually packed in aluminum foil and stored at −20 °C until further analysis. The time of filtration was measured and noted.

### 2.3. eDNA Extraction

Each filtration membrane was cut in three equal sections weighing approximately 20 mg each. The eDNA extraction was carried out as previously described [31] using the commercial DNeasy Blood & Tissue Kit (Qiagen, Hilden, Germany). To ensure that there was no cross-contamination between samples, the scissors and forceps used to cut the membrane sections were decontaminated with bleach between each sample. Moreover, a negative control membrane with no water filtration was equally processed.

### 2.4. qPCR Amplification

Each section from each membrane was individually analyzed by qPCR (StepOne, Fort Walton Beach, FL, USA, Applied Biosystems, Foster City, CA, USA), running three qPCR replicates from each one, meaning that a total of nine reactions were performed for each filtered membrane. The qPCR reactions were prepared using the Takyon™ No ROX SYBR^®^ kit (Eurogentec, Vilnius, Lithuania). Each reaction had a final volume of 10 µL containing 3.4 µL of undiluted DNA, 5 µL of MasterMix 2X, and 1.6 µL of primers mix (10 µM). The qPCR program was set using the default program provided by StepOne software v2.3 (denaturation at 95 °C for 10 min, followed by 40 cycles at 95 °C for 15 s and 60 °C for 1 min).

The filtered membrane section was considered positive for *B. truncatus* eDNA if at least one of its qPCR replicates was amplified above the limit of detection and its melting curve matched with that of its control sample. The limit of detection (LOD) was calculated as recommended [32], considering it as the lowest concentration of the standard curve dilutions (1/10) using the eDNA control with BAC for calculating the standard curve of the BAC experiment and eDNA control without BAC for calculating the standard curve of the BAC-free experiment, with at least one positive replicate of the three replicates.

The primers used to amplify *B. truncatus* eDNA were Btco1F (5′ TYGAAGGAGGGGTTGGAACA 3′) coupled with Btco1R (5′ RKTRATTCCTGGTGCYCGT 3′) [31], which amplifies 179 bp of the mitochondrial gene *cox*1. For *B. truncatus* qPCR assays, we used the following five controls: (1) positive control for *B. truncatus* DNA extracted from an adult snail (positive qPCR control, and used for the design of the standard curve); (2) positive control for *B. truncatus* eDNA: filtered membrane of 1.5 L *B. truncatus* aquarium with a density of 18 snails/L maintained in the same conditions as the other aquaria; (3) negative filtration control: filtered membrane from mineral water maintained under the same conditions as the other aquaria, but without the presence of the snails; (4) negative control of the eDNA extraction procedure: membrane with no water filtration and performed together with the tested membrane extractions; and (5) qPCR negative control: blank qPCR containing only mineral water and the qPCR MasterMix.

### 2.5. Statistical Analyses of the Laboratory Experiment

The influence of snail density and the presence of DNA preservative on eDNA concentration and decay time were evaluated by applying multivariable linear mixed regression. The “eDNA concentration” was considered as the response, while “snail density”, “presence of BAC”, and “time” were included as explanatory variables. The explanatory variable “time” was considered as a second-order polynomial. Polynomial regression is considered to be a special case of multiple linear regression, allowing to fit a non-linear relationship between the response variable and the corresponding explanatory variable. In addition, the explanatory variables were analyzed in two two-way interactions (snail density * time^2^ + BAC * time^2^) in consideration of the concomitant influence between them. The random factor “Aquaria ID” was included to account for the dependent nature of the repeated measures. Likewise, membrane “section” was also included as a random factor but nested within “sample” to take into account its lack of independence.

The relevance of the interaction terms was evaluated with the second-order AICc to account for small sample sizes [33]. When the inclusion of each interaction did not reduce AICc values in 2 or more units (ΔAICc < 2), it was dropped from the model. The main effects (whether significant or not) were retained. The normality of the distribution of the residuals of each final model was verified, and transformations were applied when necessary. Statistical analyses were performed with R Statistical Software (‘R: A language and environment for statistical computing’, version 4.2.2 [2022-10-31 ucrt], http://www.r-project.org, accessed on 27 April 2025) and RStudio (‘RStudio: Integrated development environment for R’, version 2023.06.1.524, http://www.rstudio.com/, accessed on 27 April 2025). Results were considered statistically significant when *p*-value < 0.05.

## 3. Results

### 3.1. Influence of Benzalkonium Chloride on B. truncatus eDNA Decay and eDNA Detection Sensitivity

**Figure 2:**
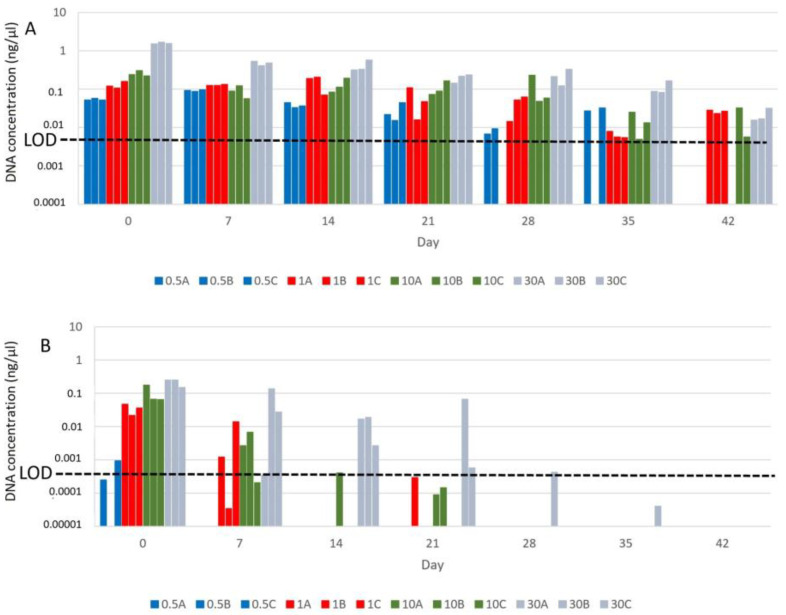
DNA concentration (ng/µL) in water samples. (**A**) Concentration (ng/µL) of *B. truncatus* eDNA obtained from the experiment using BAC (0.01%) as DNA preservative. The three replicates (A, B, C) of each snail density aquaria (0.5/L-blue, 1/L-red, 10/L-green, and 30/L-grey) are represented in the figure. LOD (0.0083 ng/µL) is shown by a dashed line. The y-axis is represented in a logarithmic scale. (**B**) Concentration (ng/µL) of *B. truncatus* eDNA obtained from the experiment without BAC addition. The three replicates (A, B, C) of each density aquaria (0.5/L-blue, 1/L-red, 10/L-green and 30/L-grey) are represented in the figure. LOD (0.00063) is shown by a dashed line. The y-axis is represented in a logarithmic scale.

Samples with BAC showed positive results until day 35 in every snail density scenario, meaning this technique can detect a vector concentration of at least 0.5 snails/L (Figure 2A). Nevertheless, one of the replicate aquaria of the lowest density (0.5 snails/L) stopped amplifying after day 21, and, by day 42, amplification was not detected in any of those aquaria (Figure 2A). Therefore, although the sensitivity decreased from day 21, the detection capacity was maintained until day 42 when adding BAC (0.01%) at densities higher than 0.5 snails/L.

These results contrast greatly with those found in the samples without BAC (Figure 2B). The eDNA from aquaria with 0.5 snail/L was amplified only after sample collection (experimental day 0), while the eDNA from the aquaria with densities of 1 snail/L and 10 snails/L was amplified up to day 7 and completely disappeared after day 21. Positive results were obtained up to day 28 at most in only one aquarium with the higher snail density (30 snails/L).

**Figure 3:**
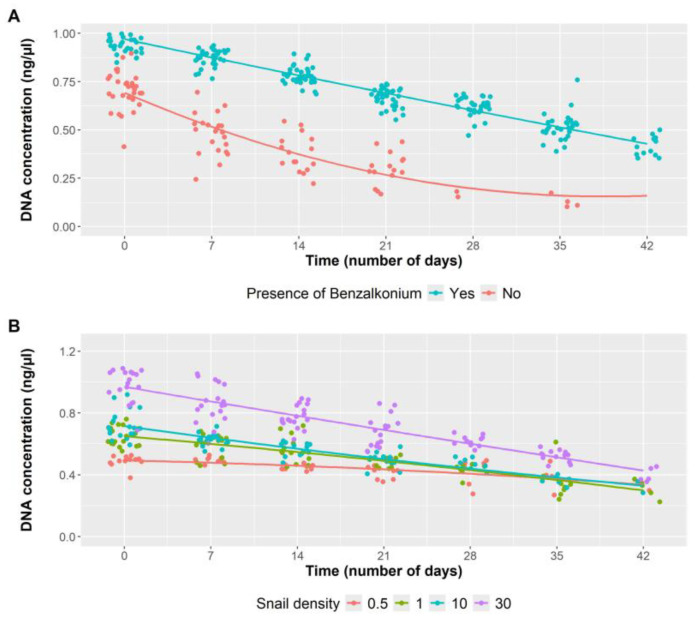
(**A**) Influence of presence/absence of BAC (0.01%) on eDNA decay. Predictions obtained from the multivariable linear mixed model displaying how the elapse of time affects the eDNA concentration. The decay kinetics of the eDNA concentration is shown using the means of the four snail densities groups (0.5/L, 1/L, 10/L, and 30/L). DNA concentration is expressed in ng/µL. The plotted points represent the partial residuals of the fitted mixed models. (**B**) Influence of snail densities (0.5/L, 1/L, 10/L, and 30/L) on eDNA detection along time. Predictions obtained from the multivariable linear mixed model displaying how the elapse of time affects the eDNA concentration. The decay kinetics of the eDNA concentration is shown using the means of both groups (with and without BAC). DNA concentration is expressed in ng/µL. The plotted points represent the partial residuals of the fitted mixed models.

Predictions obtained from the multivariable linear mixed model confirm the observations from the empirical data presented in Figure 2. An overview of the effect of adding BAC (0.01%) to the samples, regardless of the density of snails in the water, shows that the inclusion of the preservative significantly reduces the eDNA decay over time (Figure 3A and Table 1). In the presence of BAC, the eDNA concentration slightly decreased in time, while in its absence, the concentration decreased markedly after only 7 days of storage.

### 3.2. Influence of Snail Density in eDNA Detection

Our tests at different densities of snails in the water reveal that the number of positively amplified samples decreases over time at any density of snail population, and it can be observed that the samples with the lowest density (0.5 snails/L), treated or not, are the first to become undetectable (day 7 for samples without BAC and day 35 for samples with BAC) (Figure 2A,B).

**Figure 4:**
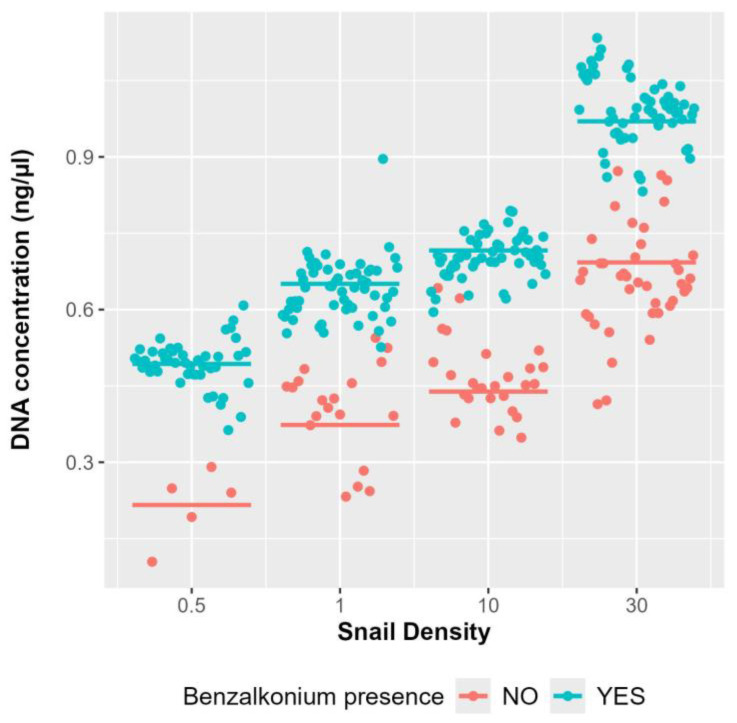
Predictions obtained from the multivariable linear mixed models displaying the influence of snail densities (0.5/L, 1/L, 10/L, and 30/L) on eDNA concentration immediately after sampling. DNA concentration is expressed in ng/µL. The plotted points represent the partial residuals of the fitted mixed models.

The density of *B. truncatus* snails present in the water has a direct effect on the concentration of eDNA present in the water samples (Figure 3B, Table 1). When considering only the concentration of snails in the water, regardless of whether it contains BAC or not, the association between eDNA concentration and snail density is evident (Figure 4). This relationship persists from day one, with eDNA concentrations of 0.5 ng/µL and 1.1 ng/µL for the lowest and highest densities, respectively, until day 35, with concentrations of 0.3 ng/µL and 0.6 ng/µL, respectively.

Finally, concerning the temperature and pH from the water samples prior to filtration, the values were the following: samples without BAC, mean temperature 22.32 °C (standard deviation 1.85) and mean pH 8.55 (standard deviation 0.19); samples with BAC, mean temperature 21.79 °C (standard deviation 1.36) and mean pH 8.43 (standard deviation 1.36).

The raw data used in this study for Figure 2, Figure 3 and Figure 4 is provided in Supplementary Table S1.

## 4. Discussion

### 4.1. Challenges and Advances in the Application of eDNA for Surveillance of Snail-Borne Parasitic Diseases

The use of eDNA as a tool for monitoring schistosomiasis and other snail-borne diseases is receiving increasing attention, as highlighted in recent studies. To date, its application for detecting parasites affecting humans and animals has primarily focused on trematode species within the genera *Schistosoma*, *Trichobilharzia*, *Opisthorchis*, and *Fasciola* [11,29,34,35,36,37,38]. These are all snail-borne parasites with free-living transmission stages, which makes them particularly suitable for detection through eDNA methodologies [38]. In addition, eDNA techniques have been used to detect disease vectors, such as snails and mosquitoes, involved in the transmission of snail-borne trematodiases and protozoan and filarial parasites, respectively [39,40]. Although eDNA is increasingly applied in environmental monitoring, its use in parasitology and disease surveillance remains in its early stages [10,38].

Common technical limitations of eDNA-based methods include contamination, false positives and negatives, eDNA degradation, and the inability to distinguish between life stages (e.g., miracidium vs. cercariae) or between living and dead organisms [27,38,41]. DNA can be released into aquatic environments through active shedding by living cells or the lysis of dead ones, enabling the detection of medically relevant vectors in water bodies [38,42].

While preventive chemotherapy remains the main strategy for schistosomiasis control, effective elimination requires an integrated approach, including vector surveillance and control [43]. eDNA offers a valuable tool for identifying risk areas without the need for direct collection of snail specimens [34,38]. It has been employed to detect eDNA of *B. truncatus* and *Oncomelania hupensis quadrasi*, vectors of urogenital and intestinal schistosomiasis, respectively [30,31]. Techniques such as qPCR, digital droplet PCR (ddPCR), and loop-mediated isothermal amplification (LAMP) have been developed to enhance field detection [31,44,45].

Species-specific primers and target genes for eDNA detection are available for key schistosome species, including *S. mansoni*, *S. haematobium*, *S. japonicum*, *S. mattheei*, and *S. bovis/S. curassoni/S. guineensis* [11,29,30,34,46,47]. For fascioliasis, eDNA of *Fasciola hepatica* has been successfully detected in its snail vectors *Galba truncatula* and *Austropeplea tomentosa* [35,48,49,50,51]. Environmental metabarcoding approaches have also proven effective for characterizing freshwater gastropod communities, providing results comparable to traditional malacological surveys [52].

Despite the clear utility of eDNA for detecting snail vectors, its application to large-scale endemic mapping remains limited. On-site DNA extraction and qPCR are particularly advantageous in time-sensitive or remote field conditions where sample degradation is a concern [25]. Several studies emphasize the importance of immediate water filtration following sample collection to preserve DNA integrity [29,30,34,35,47,48,52]. Physical and chemical factors—such as temperature, light exposure, and trophic conditions—can significantly affect eDNA stability, particularly in tropical regions lacking cold-chain infrastructure [48,53]. In our results, we did not observe significant differences in temperature and pH values between the two groups analyzed. Nevertheless, these parameters should be considered in future studies, as they may influence eDNA preservation and detection over time.

Climatic variations may further influence these degradation processes [54]. In marine ecology, eDNA has been widely used since 2008 for monitoring aquatic organisms [53]. While some agents like BAC have been shown to slow degradation, their efficacy varies and has not yet been tested for schistosomiasis vectors [28,55]. A recent field protocol proposes filtering water samples immediately and preserving filters in lysis buffer at ambient temperature to stabilize eDNA [45]. Although effective, this approach may be logistically challenging for large-scale surveillance due to the time required for immediate filtration.

### 4.2. Improved Detection of *B. truncatus* eDNA Using BAC: Implications for Field Surveillance and Sample Preservation

This study demonstrates that treating water samples with a preservative such as 0.01% BAC effectively stabilizes *B. truncatus* eDNA, allowing for methodological standardization and reducing the risk of false negatives. This is particularly relevant for comparative studies across laboratories. Preservation with BAC enables detection at low snail densities (0.5 snails/L), which is likely more representative of natural settings, and maintains eDNA detectability for up to 42 days (for densities above 0.5 snails/L). Thus, this approach addresses two key challenges in field sampling: detection at low vector densities and prevention of post-collection eDNA degradation. However, further testing under varying environmental conditions (e.g., temperature, sediment content) is necessary to confirm its applicability in the field.

Our findings confirm a positive correlation between snail density and eDNA concentration, aligning with prior observations from natural habitats [30,31]. While one previous study investigated the density of *Biomphalaria* snails and *S. mansoni* eDNA without finding a clear correlation—likely due to variability in infection prevalence and cercarial shedding [11]—our results provide more direct evidence of the link between vector abundance and environmental DNA levels. This correlation may support risk stratification by distinguishing high- from low-transmission areas, thereby informing MDA programs. Although MDA decisions involve multiple epidemiological and economic factors, beyond snail presence, eDNA detection, while not sufficient on its own to determine intervention strategies, can serve as a complementary surveillance tool to support decision-making, particularly in resource-limited settings.

In terms of efficacy and efficiency, our results show that the eDNA method reliably detects *B. truncatus* under controlled conditions, with a clear correlation between snail density and eDNA concentration. While further large-scale validation is needed, the method appears applicable to natural freshwater environments. eDNA sampling offers a rapid, cost-effective alternative to traditional surveys, particularly in remote or resource-limited settings, and could enhance the targeting of interventions such as MDA by supporting more appropriate strategic decision-making.

Given the potential range expansion of *B. truncatus* due to climate change and globalization [2,3,4,56], and the emergence of autochthonous schistosomiasis cases in Europe, monitoring the distribution of this vector is increasingly urgent. Populations previously considered non-endemic are now at risk, particularly as schistosomiasis is frequently diagnosed in migrants from endemic regions upon arrival in Europe [16,18,23,24,57,58,59,60,61,62,63,64]. Notably, the disease has already entered in Europe with an outbreak in Corsica beginning in 2013, which now appears to be becoming endemic [17,19,20,21,22]. Additionally, sporadic autochthonous cases have been reported in southern Spain [8]. In Corsica, the persistence of *B. truncatus* through winter appears to facilitate sustained transmission cycles [31].

In the framework of vector-borne diseases, where transmission depends on the presence of specific vectors, mapping their spatial distribution is essential for effective disease control [15,45,65,66]. However, the spatial and temporal variability in the distribution of freshwater snails poses significant challenges to the development of accurate risk maps [31]. The challenge of predicting future schistosomiasis outbreaks in Europe has been associated with limited data on the distribution of the snail vector [17], a gap that could potentially be addressed through the rapid and effective detection of its eDNA.

In this context, eDNA provides a rapid, accurate, and non-invasive surveillance method capable of identifying potential transmission foci, especially in previously non-endemic regions [17,18]. Our findings highlight its promise for early detection and risk mapping, critical for timely control measures.

The main strengths of this study lie in demonstrating the potential of BAC to preserve *B. truncatus* eDNA under simulated field conditions, and in identifying a positive correlation between snail density and eDNA concentration. Additionally, the study supports the use of this method for surveillance and risk assessment, while offering valuable insights into optimizing sample preservation for extended storage. Limitations include testing only one snail species and a single BAC concentration, as well as the absence of true field conditions. These factors may affect the generalizability of the results, highlighting the need for further research in the subject with validation in diverse environments.

## 5. Conclusions

In summary, the addition of BAC effectively preserves *B. truncatus* eDNA in water samples for extended periods, meeting the World Health Organization’s requirement for ensuring the stability of genetic material in field-collected samples. The incorporation of BAC also enhances the sensitivity and reproducibility of *B. truncatus* eDNA detection. Consequently, we recommend its use in eDNA-based field studies particularly in settings where immediate water filtering is not feasible, and where cold-chain logistics are limited, such as in schistosomiasis-endemic regions. These findings contribute to the advancement of reliable eDNA protocols for field use, enabling more accurate monitoring of snail populations and improved assessment of transmission risk. Finally, while our data indicate a correlation between snail density and eDNA concentration, further research is needed to assess the effectiveness and efficiency of this method under field conditions. Therefore, although eDNA-based surveillance represents a promising complementary tool to support schistosomiasis control strategies, any implications for guiding MDA should be considered preliminary until validated through broader field application and operational feasibility studies.

## Figures and Tables

**Table 1:** Final model, estimates, and *p*-values of the model explaining the association between eDNA concentration (response, ng/µL 0.25) and its predicting variables (time of collection, presence of BAC, and snail density (SnailDens)).

Final Model: eDNA ng/µl ^0.25^ ~ Snail Density × Time^2^ + BAC × Time^2^ + (1|Aquaria ID) + (1|Sample/Section)			
Predictors	Estimates	CI	*p*-Value
Intercept	0.09	0.04–0.14	**0.001**
BAC [YES]	0.36	0.33–0.39	**<0.001**
Time [1^st^ degree]	−1.13	−2.10–−0.16	**0.022**
Time [2^nd^ degree]	0.78	−0.13–1.69	0.094
SnailDens [1]	0.08	0.02–0.14	**0.006**
SnailDens [10]	0.11	0.05–0.17	**<0.001**
SnailDens [30]	0.32	0.26–0.37	**<0.001**
BAC [YES] × Time [1^st^ degree]	0.37	−0.24–0.98	0.232
BAC [YES] × Time [2^nd^ degree]	−0.90	−1.42–−0.38	**0.001**
SnailDens [1] × Time [1^st^ degree]	−1.03	−2.03–−0.04	**0.042**
SnailDens [1] × Time [2^nd^ degree]	0.03	−0.94–0.99	0.958
SnailDens [10] × Time [1^st^ degree]	−1.32	−2.33–−0.30	**0.011**
SnailDens [10] × Time [2^nd^ degree]	0.25	−0.76–1.25	0.627
SnailDens [30] × Time [1^st^ degree]	−2.10	−3.10–−1.10	**<0.001**
SnailDens [30] × Time [2^nd^ degree]	0.17	−0.80–1.13	0.730

Significant *p*-values are marked in bold.

## Data Availability

The original contributions presented in this study are included in the article and supplementary material. Further inquiries can be directed to the corresponding authors.

## Supplementary Materials

The following supporting information can be downloaded at: https://www.mdpi.com/article/10.3390/tropicalmed10080201/s1. Figure S1: *B. truncatus* snails from Almería, Spain. (A) Dorsal and ventral views of shells. (B) Live specimen from El Ejido, Almería (shell length 8.9 mm). (C) Live specimens born and reared under laboratory conditions. Table S1: Raw data used in this study for Figure 2, Figure 3 and Figure 4.

## Abbreviations

The following abbreviations are used in this manuscript:

AICc	Corrected Akaike Information Criterion
BAC	Benzalkonium chloride
eDNA	Environmental DNA
LOD	Limit of detection
MDA	Mass drug administration
NTD	Neglected tropical diseases
WHO	World Health Organization
qPCR	Quantitative polymerase chain reaction
ddPCR	Digital droplet polymerase chain reaction
LAMP	Loop-mediated isothermal amplification

## References

[1] Vos T., Abajobir A.A., Abate K.H., Abbafati C., Abbas K.M., Abd-Allah F., Abdulkader R.S., Abdulle A.M., Abebo T.A., Abera S.F. (2017). Global, Regional, and National Incidence, Prevalence, and Years Lived with Disability for 328 Diseases and Injuries for 195 Countries, 1990–2016: A Systematic Analysis for the Global Burden of Disease Study 2016. Lancet.

[2] Martínez-Ortí A., Adam S., Garippa G., Boissier J., Bargues M.D., Mas-Coma S. (2022). Morpho-Anatomical Characterization of the Urogenital Schistosmiasis Vector *Bulinus truncatus* (Audouin, 1827) (Heterobranchia: Bulinidae) from Southwestern Europe. J. Conchol..

[3] Mas-Coma S., Valero M.A., Bargues M.D. (2009). Climate Change Effects on Trematodiases, with Emphasis on Zoonotic Fascioliasis and Schistosomiasis. Vet. Parasitol..

[4] van der Deure T., Maes T., Huyse T., Stensgaard A.-S. (2024). Climate Change Could Fuel Urinary Schistosomiasis Transmission in Africa and Europe. Glob. Change Biol..

[5] World Health Organization (WHO) Ending the Neglect to Attain the Sustainable Development Goals: A Road Map for Neglected Tropical Diseases 2021–2030. https://www.who.int/publications-detail-redirect/9789240010352.

[6] Mas-Coma S., Valero M.A., Bargues M.D. (2023). One Health for Fascioliasis Control in Human Endemic Areas. Trends Parasitol..

[7] Ramalli L., Mulero S., Noël H., Chiappini J.-D., Vincent J., Barré-Cardi H., Malfait P., Normand G., Busato F., Gendrin V. (2018). Persistence of Schistosomal Transmission Linked to the Cavu River in Southern Corsica since 2013. Eurosurveillance.

[8] Salas-Coronas J., Bargues M.D., Lozano-Serrano A.B., Artigas P., Martínez-Ortí A., Mas-Coma S., Merino-Salas S., Abad Vivas-Pérez J.I. (2021). Evidence of Autochthonous Transmission of Urinary Schistosomiasis in Almeria (Southeast Spain): An Outbreak Analysis. Travel Med. Infect. Dis..

[9] Stothard J.R., Campbell S.J., Osei-Atweneboana M.Y., Durant T., Stanton M.C., Biritwum N.-K., Rollinson D., Ombede D.R.E., Tchuem-Tchuenté L.-A. (2017). Towards Interruption of Schistosomiasis Transmission in Sub-Saharan Africa: Developing an Appropriate Environmental Surveillance Framework to Guide and to Support “end Game” Interventions. Infect. Dis. Poverty.

[10] Selbach C., Jorge F., Dowle E., Bennett J., Chai X., Doherty J.-F., Eriksson A., Filion A., Hay E., Herbison R. (2019). Parasitological Research in the Molecular Age. Parasitology.

[11] Sengupta M.E., Hellström M., Kariuki H.C., Olsen A., Thomsen P.F., Mejer H., Willerslev E., Mwanje M.T., Madsen H., Kristensen T.K. (2019). Environmental DNA for Improved Detection and Environmental Surveillance of Schistosomiasis. Proc. Natl. Acad. Sci. USA.

[12] Kamel B., Laidemitt M.R., Lu L., Babbitt C., Weinbaum O.L., Mkoji G.M., Loker E.S. (2021). Detecting and Identifying *Schistosoma* Infections in Snails and Aquatic Habitats: A Systematic Review. PLoS Negl. Trop. Dis..

[13] Mas-Coma S. (2020). Human Fascioliasis Emergence Risks in Developed Countries: From Individual Patients and Small Epidemics to Climate and Global Change Impacts. Enfermedades Infecc. Microbiol. Clín. (Engl. Ed.).

[14] El-Sayed A., Kamel M. (2020). Climatic Changes and Their Role in Emergence and Re-Emergence of Diseases. Environ. Sci. Pollut. Res. Int..

[15] Cuervo P.F., Bargues M.D., Artigas P., Buchon P., Angles R., Mas-Coma S. (2024). Global Warming Induced Spread of the Highest Human Fascioliasis Hyperendemic Area. Parasit. Vectors.

[16] Beltrame A., Buonfrate D., Gobbi F., Angheben A., Marchese V., Monteiro G.B., Bisoffi Z. (2017). The Hidden Epidemic of Schistosomiasis in Recent African Immigrants and Asylum Seekers to Italy. Eur. J. Epidemiol..

[17] Kincaid-Smith J., Rey O., Toulza E., Berry A., Boissier J. (2017). Emerging Schistosomiasis in Europe: A Need to Quantify the Risks. Trends Parasitol..

[18] Gabrielli A.F., Garba Djirmay A. (2023). Schistosomiasis in Europe. Curr. Trop. Med. Rep..

[19] Boissier J., Moné H., Mitta G., Bargues M.D., Molyneux D., Mas-Coma S. (2015). Schistosomiasis Reaches Europe. Lancet Infect. Dis..

[20] Boissier J., Grech-Angelini S., Webster B.L., Allienne J.-F., Huyse T., Mas-Coma S., Toulza E., Barré-Cardi H., Rollinson D., Kincaid-Smith J. (2016). Outbreak of Urogenital Schistosomiasis in Corsica (France): An Epidemiological Case Study. Lancet Infect. Dis..

[21] Rothe C., Zimmer T., Schunk M., Wallrauch C., Helfrich K., Gültekin F., Bretzel G., Allienne J.-F., Boissier J. (2021). Developing Endemicity of Schistosomiasis, Corsica, France. Emerg. Infect. Dis..

[22] Wellinghausen N., Moné H., Mouahid G., Nebel A., Tappe D., Gabriel M. (2022). A Family Cluster of Schistosomiasis Acquired in Solenzara River, Corsica (France)—Solenzara River Is Clearly a Transmission Site for Schistosomiasis in Corsica. Parasitol. Res..

[23] De Elías-Escribano A., Artigas P., Salas-Coronas J., Luzon-Garcia M.P., Reguera-Gomez M., Cabeza-Barrera M.I., Vázquez-Villegas J., Boissier J., Mas-Coma S., Bargues M.D. (2025). *Schistosoma mansoni* x *S. haematobium* Hybrids Frequently Infecting Sub-Saharan Migrants in Southeastern Europe: Egg DNA Genotyping Assessed by RD-PCR, Sequencing and Cloning. PLoS Negl. Trop. Dis..

[24] De Elias-Escribano A., Artigas P., Salas-Coronas J., Luzon-Garcia M.P., Reguera-Gomez M., Sanchez-Marques R., Salvador F., Boissier J., Mas-Coma S., Bargues M.D. (2025). Imported Schistosomiasis in Southwestern Europe: Wide Variation of Pure and Hybrid Genotypes Infecting Sub-Saharan Migrants. Transbound. Emerg. Dis..

[25] Veilleux H.D., Misutka M.D., Glover C.N. (2021). Environmental DNA and Environmental RNA: Current and Prospective Applications for Biological Monitoring. Sci. Total Environ..

[26] Hansen B.K., Jacobsen M.W., Middelboe A.L., Preston C.M., Marin R., Bekkevold D., Knudsen S.W., Møller P.R., Nielsen E.E. (2020). Remote, Autonomous Real-Time Monitoring of Environmental DNA from Commercial Fish. Sci. Rep..

[27] Beng K.C., Corlett R.T. (2020). Applications of Environmental DNA (eDNA) in Ecology and Conservation: Opportunities, Challenges and Prospects. Biodivers. Conserv..

[28] Yamanaka H., Minamoto T., Matsuura J., Sakurai S., Tsuji S., Motozawa H., Hongo M., Sogo Y., Kakimi N., Teramura I. (2017). A Simple Method for Preserving Environmental DNA in Water Samples at Ambient Temperature by Addition of Cationic Surfactant. Limnology.

[29] Sato M.O., Rafalimanantsoa A., Ramarokoto C., Rahetilahy A.M., Ravoniarimbinina P., Kawai S., Minamoto T., Sato M., Kirinoki M., Rasolofo V. (2018). Usefulness of Environmental DNA for Detecting *Schistosoma mansoni* Occurrence Sites in Madagascar. Int. J. Infect. Dis..

[30] Fornillos R.J.C., Sato M.O., Tabios I.K.B., Sato M., Leonardo L.R., Chigusa Y., Minamoto T., Kikuchi M., Legaspi E.R., Fontanilla I.K.C. (2019). Detection of *Schistosoma japonicum* and *Oncomelania hupensis* Quadrasi Environmental DNA and Its Potential Utility to Schistosomiasis Japonica Surveillance in the Philippines. PLoS ONE.

[31] Mulero S., Boissier J., Allienne J.-F., Quilichini Y., Foata J., Pointier J.-P., Rey O. (2020). Environmental DNA for Detecting *Bulinus truncatus*: A New Environmental Surveillance Tool for Schistosomiasis Emergence Risk Assessment. Environ. DNA.

[32] Ellison S.L.R., English C.A., Burns M.J., Keer J.T. (2006). Routes to Improving the Reliability of Low Level DNA Analysis Using Real-Time PCR. BMC Biotechnol..

[33] Johnson J.B., Omland K.S. (2004). Model Selection in Ecology and Evolution. Trends Ecol. Evol..

[34] Alzaylaee H., Collins R.A., Rinaldi G., Shechonge A., Ngatunga B., Morgan E.R., Genner M.J. (2020). *Schistosoma* Species Detection by Environmental DNA Assays in African Freshwaters. PLoS Negl. Trop. Dis..

[35] Jones R.A., Brophy P.M., Davis C.N., Davies T.E., Emberson H., Rees Stevens P., Williams H.W. (2018). Detection of *Galba truncatula*, *Fasciola hepatica* and *Calicophoron daubneyi* Environmental DNA within Water Sources on Pasture Land, a Future Tool for Fluke Control?. Parasit. Vectors.

[36] Rudko S.P., Turnbull A., Reimink R.L., Froelich K., Hanington P.C. (2019). Species-Specific qPCR Assays Allow for High-Resolution Population Assessment of Four Species Avian Schistosome That Cause Swimmer’s Itch in Recreational Lakes. Int. J. Parasitol. Parasites Wildl..

[37] Hashizume H., Sato M., Sato M.O., Ikeda S., Yoonuan T., Sanguankiat S., Pongvongsa T., Moji K., Minamoto T. (2017). Application of Environmental DNA Analysis for the Detection of *Opisthorchis viverrini* DNA in Water Samples. Acta Trop..

[38] Sengupta M.E., Lynggaard C., Mukaratirwa S., Vennervald B.J., Stensgaard A.S. (2022). Environmental DNA in Human and Veterinary Parasitology—Current Applications and Future Prospects for Monitoring and Control. Food Waterborne Parasitol..

[39] Krol L., Van der Hoorn B., Gorsich E.E., Trimbos K., van Bodegom P.M., Schrama M. (2019). How Does eDNA Compare to Traditional Trapping? Detecting Mosquito Communities in South-African Freshwater Ponds. Front. Ecol. Evol..

[40] Schneider J., Valentini A., Dejean T., Montarsi F., Taberlet P., Glaizot O., Fumagalli L. (2016). Detection of Invasive Mosquito Vectors Using Environmental DNA (eDNA) from Water Samples. PLoS ONE.

[41] Harper L.R., Buxton A.S., Rees H.C., Bruce K., Brys R., Halfmaerten D., Read D.S., Watson H.V., Sayer C.D., Jones E.P. (2019). Prospects and Challenges of Environmental DNA (eDNA) Monitoring in Freshwater Ponds. Hydrobiologia.

[42] Power H., Takahashi M., Jarman S., Berry O. (2023). What Is Environmental DNA?. Environ. DNA.

[43] McManus D.P., Gordon C., Weerakoon K.G.A.D. (2018). Testing of Water Samples for Environmental DNA as a Surveillance Tool to Assess the Risk of Schistosome Infection in a Locality. Int. J. Infect. Dis..

[44] Calata F.I.C., Caranguian C.Z., Mendoza J.E.M., Fornillos R.J.C., Tabios I.K.B., Fontanilla I.K.C., Leonardo L.R., Sunico L.S., Kawai S., Chigusa Y. (2019). Analysis of Environmental DNA and Edaphic Factors for the Detection of the Snail Intermediate Host Oncomelania Hupensis Quadrasi. Pathogens.

[45] Blin M., Senghor B., Boissier J., Mulero S., Rey O., Portela J. (2023). Development of Environmental Loop-Mediated Isothermal Amplification (eLAMP) Diagnostic Tool for *Bulinus truncatus* Field Detection. Parasit. Vectors.

[46] Schols R., Carolus H., Hammoud C., Mulero S., Mudavanhu A., Huyse T. (2019). A Rapid Diagnostic Multiplex PCR Approach for Xenomonitoring of Human and Animal Schistosomiasis in a “One Health” Context. Trans. R. Soc. Trop. Med. Hyg..

[47] Alzaylaee H., Collins R.A., Shechonge A., Ngatunga B.P., Morgan E.R., Genner M.J. (2020). Environmental DNA-Based Xenomonitoring for Determining *Schistosoma* Presence in Tropical Freshwaters. Parasit. Vectors.

[48] Jones R.A., Davis C.N., Jones D.L., Tyson F., Davies E., Cutress D., Brophy P.M., Rose M.T., Williams M., Williams H.W. (2021). Temporal Dynamics of Trematode Intermediate Snail Host Environmental DNA in Small Water Body Habitats. Parasitology.

[49] Davis C.N., Tyson F., Cutress D., Davies E., Jones D.L., Brophy P.M., Prescott A., Rose M.T., Williams M., Williams H.W. (2020). Rapid Detection of *Galba truncatula* in Water Sources on Pasture-Land Using Loop-Mediated Isothermal Amplification for Control of Trematode Infections. Parasit. Vectors.

[50] Rathinasamy V., Hosking C., Tran L., Kelley J., Williamson G., Swan J., Elliott T., Rawlin G., Beddoe T., Spithill T.W. (2018). Development of a Multiplex Quantitative PCR Assay for Detection and Quantification of DNA from *Fasciola hepatica* and the Intermediate Snail Host, *Austropeplea tomentosa*, in Water Samples. Vet. Parasitol..

[51] Rathinasamy V., Tran L., Swan J., Kelley J., Hosking C., Williamson G., Knowles M., Elliott T., Rawlin G., Spithill T.W. (2021). Towards Understanding the Liver Fluke Transmission Dynamics on Farms: Detection of Liver Fluke Transmitting Snail and Liver Fluke-Specific Environmental DNA in Water Samples from an Irrigated Dairy Farm in Southeast Australia. Vet. Parasitol..

[52] Mulero S., Toulza E., Loisier A., Zimmerman M., Allienne J.-F., Foata J., Quilichini Y., Pointier J.-P., Rey O., Boissier J. (2021). Malacological Survey in a Bottle of Water: A Comparative Study between Manual Sampling and Environmental DNA Metabarcoding Approaches. Glob. Ecol. Conserv..

[53] Ficetola G.F., Miaud C., Pompanon F., Taberlet P. (2008). Species Detection Using Environmental DNA from Water Samples. Biol. Lett..

[54] Eichmiller J.J., Best S.E., Sorensen P.W. (2016). Effects of Temperature and Trophic State on Degradation of Environmental DNA in Lake Water. Environ. Sci. Technol..

[55] Takahara T., Taguchi J., Yamagishi S., Doi H., Ogata S., Yamanaka H., Minamoto T. (2020). Suppression of Environmental DNA Degradation in Water Samples Associated with Different Storage Temperature and Period Using Benzalkonium Chloride. Limnol. Ocean. Methods.

[56] Martínez-Ortí A., Vilavella D., Bargues M.D., Mas–Coma S. (2019). Risk Map of Transmission of Urogenital Schistosomiasis by *Bulinus truncatus* (Audouin, 1827) (Mollusca Gastropoda, Bulinidae) in Spain and Portugal. Anim. Biodiv. Conserv..

[57] Guido G., Frallonardo L., Cotugno S., De Vita E., Patti G., De Santis L., Segala F.V., Nicastri E., Gobbi F., Morea A. (2025). Prevalence of Neglected Tropical Diseases among Migrants Living in Europe: A Systematic Review and Meta-Analysis. Travel Med. Infect. Dis..

[58] Salvador F., Bocanegra C., Treviño B., Sulleiro E., Sánchez-Montalvá A., Serre-Delcor N., Bosch-Nicolau P., Aznar M.L., Goterris L., Pou D. (2024). Imported Schistosomiasis in Travelers: Experience from a Referral Tropical Medicine Unit in Barcelona, Spain. Travel Med. Infect. Dis..

[59] Depaquit J., Akhoundi M., Haouchine D., Mantelet S., Izri A. (2019). No Limit in Interspecific Hybridization in Schistosomes: Observation from a Case Report. Parasite.

[60] Le Govic Y., Kincaid-Smith J., Allienne J.-F., Rey O., de Gentile L., Boissier J. (2019). *Schistosoma haematobium–Schistosoma mansoni* Hybrid Parasite in Migrant Boy, France, 2017. Emerg. Infect. Dis..

[61] Salas-Coronas J., Cabezas-Fernández M.T., Lozano-Serrano A.B., Soriano-Pérez M.J., Vázquez-Villegas J., Cuenca-Gómez J.Á. (2018). Newly Arrived African Migrants to Spain: Epidemiology and Burden of Disease. Am. J. Trop. Med. Hyg..

[62] Salas-Coronas J., Vázquez-Villegas J., Lozano-Serrano A.B., Soriano-Pérez M.J., Cabeza-Barrera I., Cabezas-Fernández M.T., Villarejo-Ordóñez A., Sánchez-Sánchez J.C., Abad Vivas-Pérez J.I., Vázquez-Blanc S. (2020). Severe Complications of Imported Schistosomiasis, Spain: A Retrospective Observational Study. Travel. Med. Infect. Dis..

[63] Soentjens P., Cnops L., Huyse T., Yansouni C., De Vos D., Bottieau E., Clerinx J., Van Esbroeck M. (2016). Diagnosis and Clinical Management of *Schistosoma haematobium*—*Schistosoma bovis* Hybrid Infection in a Cluster of Travelers Returning from Mali. Clin. Infect. Dis..

[64] Cnops L., Huyse T., Maniewski U., Soentjens P., Bottieau E., Van Esbroeck M., Clerinx J. (2021). Acute Schistosomiasis with a *Schistosoma mattheei × Schistosoma haematobium* Hybrid Species in a Cluster of 34 Travelers Infected in South Africa. Clin. Infect. Dis..

[65] Cuervo P.F., Artigas P., Lorenzo-Morales J., Bargues M.D., Mas-Coma S. (2023). Ecological Niche Modelling Approaches: Challenges and Applications in Vector-Borne Diseases. Trop. Med. Infect. Dis..

[66] Cuervo P.F., Bargues M.D., Artigas P., Buchon P., Angles R., Mas-Coma S. (2025). Heterogeneous Zonal Impacts of Climate Change on a Wide Hyperendemic Area of Human and Animal Fascioliasis Assessed within a One Health Action for Prevention and Control. PLoS Negl. Trop. Dis..

