# Supplementary material for: Benzalkonium Chloride Significantly Improves Environmental DNA Detection from Schistosomiasis Snail Vectors in Freshwater Samples
Source: Trop Med Infect Dis. 2025 Jul 22;10(8):201. doi: 10.3390/tropicalmed10080201 (PMC12390305; doi:10.3390/tropicalmed10080201)
Supplement: Supplementary file 1 [file tropicalmed-10-00201-s001.zip › tropicalmed-3662214 Table S1.pdf]

Table S1. Raw data used in the analyses of the research article entitled "Chloride benzalkonium significantly improves environmental DNA detection from schistosomiasis snail vectors in freshwater samples".

id	time	snail_density	aquarium_id	sample	BAC_presence	section	ct	eDNA_concentration
1	0	0.5	A	0.0.5.A1	YES	1	29.921	0.049568254
2	0	0.5	A	0.0.5.A1	YES	2	30.018	0.047604396
3	0	0.5	A	0.0.5.A1	YES	3	29.562	0.057567981
4	0	0.5	B	0.0.5.B1	YES	1	29.721	0.053876914
5	0	0.5	B	0.0.5.B1	YES	2	29.447	0.060394234
6	0	0.5	B	0.0.5.B1	YES	3	29.633	0.055889512
7	0	0.5	C	0.0.5.C1	YES	1	30.168	0.044719582
8	0	0.5	C	0.0.5.C1	YES	2	29.375	0.062233919
9	0	0.5	C	0.0.5.C1	YES	3	29.91	0.049796013
10	0	1	D	0.1.D1	YES	1	27.592	0.130840466
11	0	1	D	0.1.D1	YES	2	27.649	0.127768954
12	0	1	D	0.1.D1	YES	3	28.187	0.10210565
13	0	1	E	0.1.E1	YES	1	28.084	0.106584072
14	0	1	E	0.1.E1	YES	2	27.828	0.118584288
15	0	1	E	0.1.E1	YES	3	28.442	0.091811256
16	0	1	F	0.1.F1	YES	1	27.072	0.162502381
17	0	1	F	0.1.F1	YES	2	27.275	0.149319885
18	0	1	F	0.1.F1	YES	3	27.065	0.162977142
19	0	10	G	0.10.G1	YES	1	25.886	0.266391499
20	0	10	G	0.10.G1	YES	2	26.436	0.211822871
21	0	10	G	0.10.G1	YES	3	26.089	0.244781325
22	0	10	H	0.10.H1	YES	1	25.489	0.314322569
23	0	10	H	0.10.H1	YES	2	25.671	0.291362993
24	0	10	H	0.10.H1	YES	3	25.565	0.304522867
25	0	10	I	0.10.I1	YES	1	26.316	0.222685689
26	0	10	I	0.10.I1	YES	2	26.36	0.21863944
27	0	10	I	0.10.I1	YES	3	26.361	0.21854834
28	0	30	J	0.30.J1	YES	1	22.174	1.251340687
29	0	30	J	0.30.J1	YES	2	21.442	1.697712919
30	0	30	J	0.30.J1	YES	3	21.566	1.612207045
31	0	30	K	0.30.K1	YES	1	21.506	1.653029268
32	0	30	K	0.30.K1	YES	2	21.559	1.616917207
33	0	30	K	0.30.K1	YES	3	21.44	1.699128578
34	0	30	L	0.30.L1	YES	1	21.571	1.608851046
35	0	30	L	0.30.L1	YES	2	21.653	1.554798841
36	0	30	L	0.30.L1	YES	3	21.8	1.462405707
37	7	0.5	A	7.0.5.A1	YES	1	29.027	0.071947147
38	7	0.5	A	7.0.5.A1	YES	2	27.893	0.11541505
39	7	0.5	A	7.0.5.A1	YES	3	28.389	0.093861759
40	7	0.5	B	7.0.5.B1	YES	1	28.683	0.083037835
41	7	0.5	B	7.0.5.B1	YES	2	28.701	0.082417246
42	7	0.5	B	7.0.5.B1	YES	3	28.325	0.096398971
43	7	0.5	C	7.0.5.C1	YES	1	28.272	0.098551936
44	7	0.5	C	7.0.5.C1	YES	2	28.667	0.083593392
45	7	0.5	C	7.0.5.C1	YES	3	28.153	0.103562761
46	7	1	D	7.1.D1	YES	1	27.523	0.134657569
47	7	1	D	7.1.D1	YES	2	27.72	0.124043685
48	7	1	D	7.1.D1	YES	3	27.974	0.11158397
49	7	1	E	7.1.E1	YES	1	27.525	0.134545376

50	7	1	E	7.1.E1	YES	2	27.696	0.125290618
51	7	1	E	7.1.E1	YES	3	27.949	0.112752634
52	7	1	F	7.1.F1	YES	1	27.743	0.122860355
53	7	1	F	7.1.F1	YES	2	27.437	0.139571382
54	7	1	F	7.1.F1	YES	3	27.52	0.134826033
55	7	10	G	7.10.G1	YES	1	28.62	0.085246927
56	7	10	G	7.10.G1	YES	2	28.489	0.090030392
57	7	10	G	7.10.G1	YES	3	28.538	0.088210516
58	7	10	H	7.10.H1	YES	1	27.557	0.132762959
59	7	10	H	7.10.H1	YES	2	27.549	0.133206337
60	7	10	H	7.10.H1	YES	3	28.216	0.100879032
61	7	10	I	7.10.I1	YES	1	28.486	0.090143025
62	7	10	I	7.10.I1	YES	2	29.352	0.062833325
63	7	10	I	7.10.I1	YES	3	31.006	0.031537229
64	7	30	J	7.30.J1	YES	1	24.029	0.57760236
65	7	30	J	7.30.J1	YES	2	24.434	0.487894353
66	7	30	J	7.30.J1	YES	3	24.275	0.521319623
67	7	30	K	7.30.K1	YES	1	24.602	0.454902739
68	7	30	K	7.30.K1	YES	2	24.854	0.409550719
69	7	30	K	7.30.K1	YES	3	25.175	0.358268104
70	7	30	L	7.30.L1	YES	1	24.855	0.409380071
71	7	30	L	7.30.L1	YES	2	24.623	0.450938844
72	7	30	L	7.30.L1	YES	3	24.771	0.423965304
73	14	0.5	A	14.0.5.A1	YES	1	31.911	0.026854347
74	14	0.5	A	14.0.5.A1	YES	2	30.712	0.044443701
75	14	0.5	A	14.0.5.A1	YES	3	31.026	0.038950313
76	14	0.5	B	14.0.5.B1	YES	1	32.12	0.024596647
77	14	0.5	B	14.0.5.B1	YES	2	31.742	0.028830622
78	14	0.5	B	14.0.5.B1	YES	3	32.127	0.024524408
79	14	0.5	C	14.0.5.C1	YES	1	31.927	0.026674414
80	14	0.5	C	14.0.5.C1	YES	2	31.282	0.034978052
81	14	0.5	C	14.0.5.C1	YES	3	31.932	0.026618433
82	14	1	D	14.1.D1	YES	1	27.517	0.170152924
83	14	1	D	14.1.D1	YES	2	27.434	0.176191682
84	14	1	D	14.1.D1	YES	3	27.674	0.159290465
85	14	1	E	14.1.E1	YES	1	26.909	0.219678888
86	14	1	E	14.1.E1	YES	2	27.334	0.183752635
87	14	1	E	14.1.E1	YES	3	27.815	0.150127362
88	14	1	F	14.1.F1	YES	1	29.858	0.0636282
89	14	1	F	14.1.F1	YES	2	30.583	0.046919175
90	14	1	F	14.1.F1	YES	3	29.672	0.068800444
91	14	10	G	14.10.G1	YES	1	29.046	0.089500367
92	14	10	G	14.10.G1	YES	2	29.999	0.05996802
93	14	10	G	14.10.G1	YES	3	29.83	0.064381208
94	14	10	H	14.10.H1	YES	1	28.811	0.098788873
95	14	10	H	14.10.H1	YES	2	28.505	0.112343369
96	14	10	H	14.10.H1	YES	3	29.29	0.080779148
97	14	10	I	14.10.I1	YES	1	27.337	0.183521154
98	14	10	I	14.10.I1	YES	2	27.602	0.164183106
99	14	10	I	14.10.I1	YES	3	27.495	0.171733102
100	14	30	J	14.30.J1	YES	1	26.234	0.291718186
101	14	30	J	14.30.J1	YES	2	26.206	0.295170528
102	14	30	J	14.30.J1	YES	3	26.264	0.288064043

103	14	30	K	14.30.K1	YES	1	26.279		0.286254172
104	14	30	K	14.30.K1	YES	2	25.869		0.3400708
105	14	30	K	14.30.K1	YES	3	26.239		0.291105959
106	14	30	L	14.30.L1	YES	1	24.75		0.544209882
107	14	30	L	14.30.L1	YES	2	24.675		0.561632918
108	14	30	L	14.30.L1	YES	3	24.957		0.498876103
109	21	0.5	A	21.0.5.A1	YES	1	32.59		0.020188753
110	21	0.5	A	21.0.5.A1	YES	2	32.108		0.024720981
111	21	0.5	A	21.0.5.A1	YES	3	34.31		0.009800334
112	21	0.5	B	21.0.5.B1	YES	1	33.129		0.016097255
113	21	0.5	B	21.0.5.B1	YES	2	34.732		0.008207929
114	21	0.5	B	21.0.5.B1	YES	3	NA	NA	
115	21	0.5	C	21.0.5.C1	YES	1	30.799		0.04284837
116	21	0.5	C	21.0.5.C1	YES	2	30.378		0.051139812
117	21	0.5	C	21.0.5.C1	YES	3	32.484		0.021108266
118	21	1	D	21.1.D1	YES	1	28.96		0.092793648
119	21	1	D	21.1.D1	YES	2	28.496		0.112769013
120	21	1	D	21.1.D1	YES	3	29.427		0.076260434
121	21	1	E	21.1.E1	YES	1	33.546		0.013510049
122	21	1	E	21.1.E1	YES	2	34.091		0.01074495
123	21	1	E	21.1.E1	YES	3	33.818		0.012050967
124	21	1	F	21.1.F1	YES	1	30.43		0.050034559
125	21	1	F	21.1.F1	YES	2	31.833		0.027749054
126	21	1	F	21.1.F1	YES	3	30.949		0.040231113
127	21	10	G	21.10.G1	YES	1	30.089		0.057742602
128	21	10	G	21.10.G1	YES	2	29.713		0.067625344
129	21	10	G	21.10.G1	YES	3	30.108		0.057283454
130	21	10	H	21.10.H1	YES	1	29.257		0.081907028
131	21	10	H	21.10.H1	YES	2	29.307		0.080204194
132	21	10	H	21.10.H1	YES	3	29.741		0.066834392
133	21	10	I	21.10.I1	YES	1	32.871		0.0179404
134	21	10	I	21.10.I1	YES	2	33.098		0.016308303
135	21	10	I	21.10.I1	YES	3	17.679		10.61927556
136	21	30	J	21.30.J1	YES	1	28.772		0.100421064
137	21	30	J	21.30.J1	YES	2	28.103		0.133016375
138	21	30	J	21.30.J1	YES	3	27.817		0.150001254
139	21	30	K	21.30.K1	YES	1	27.014		0.210197592
140	21	30	K	21.30.K1	YES	2	27.127		0.200450572
141	21	30	K	21.30.K1	YES	3	27.474		0.173255141
142	21	30	L	21.30.L1	YES	1	26.853		0.224909241
143	21	30	L	21.30.L1	YES	2	26.897		0.22078934
144	21	30	L	21.30.L1	YES	3	27.181		0.195953617
145	28	0.5	A	28.0.5.A1	YES	1	35.981		0.005711784
146	28	0.5	A	28.0.5.A1	YES	2	NA	NA	
147	28	0.5	A	28.0.5.A1	YES	3	NA	NA	
148	28	0.5	B	28.0.5.B1	YES	1	NA	NA	
149	28	0.5	B	28.0.5.B1	YES	2	32.146		0.029395076
150	28	0.5	B	28.0.5.B1	YES	3	38.219		0.002195626
151	28	0.5	C	28.0.5.C1	YES	1	27.516		0.212457823
152	28	0.5	C	28.0.5.C1	YES	2	27.314		0.231605856
153	28	0.5	C	28.0.5.C1	YES	3	27.79		0.188989575
154	28	1	D	28.1.D1	YES	1	32.677		0.023429273
155	28	1	D	28.1.D1	YES	2	NA	NA	

156	28	1	D	28.1.D1	YES	3	35.53		0.006925406
157	28	1	E	28.1.E1	YES	1	30.289		0.064983258
158	28	1	E	28.1.E1	YES	2	30.901		0.050033154
159	28	1	E	28.1.E1	YES	3	31.242		0.043250644
160	28	1	F	28.1.F1	YES	1	30.636		0.056030307
161	28	1	F	28.1.F1	YES	2	30.226		0.066755922
162	28	1	F	28.1.F1	YES	3	30.161		0.068635557
163	28	10	G	28.10.G1	YES	1	27.243		0.238738289
164	28	10	G	28.10.G1	YES	2	26.946		0.271034215
165	28	10	G	28.10.G1	YES	3	26.971		0.268155
166	28	10	H	28.10.H1	YES	1	31.181		0.044392523
167	28	10	H	28.10.H1	YES	2	NA	NA	
168	28	10	H	28.10.H1	YES	3	30.812		0.051972062
169	28	10	I	28.10.I1	YES	1	31.275		0.042645197
170	28	10	I	28.10.I1	YES	2	30.249		0.066103223
171	28	10	I	28.10.I1	YES	3	30.064		0.071539427
172	28	30	J	28.30.J1	YES	1	27.243		0.238738289
173	28	30	J	28.30.J1	YES	2	27.542		0.210111092
174	28	30	J	28.30.J1	YES	3	26.924		0.273593485
175	28	30	K	28.30.K1	YES	1	28.607		0.133309424
176	28	30	K	28.30.K1	YES	2	28.773		0.124183267
177	28	30	K	28.30.K1	YES	3	28.554		0.136362162
178	28	30	L	28.30.L1	YES	1	25.828		0.436963608
179	28	30	L	28.30.L1	YES	2	26.223		0.369114758
180	28	30	L	28.30.L1	YES	3	26.332		0.352321199
181	35	0.5	A	35.0.5.A1	YES	1	32.096		0.030029702
182	35	0.5	A	35.0.5.A1	YES	2	35.292		0.00766657
183	35	0.5	A	35.0.5.A1	YES	3	30.102		0.070387471
184	35	0.5	B	35.0.5.B1	YES	1	NA	NA	
185	35	0.5	B	35.0.5.B1	YES	2	NA	NA	
186	35	0.5	B	35.0.5.B1	YES	3	NA	NA	
187	35	0.5	C	35.0.5.C1	YES	1	NA	NA	
188	35	0.5	C	35.0.5.C1	YES	2	NA	NA	
189	35	0.5	C	35.0.5.C1	YES	3	32.004		0.031233429
190	35	1	D	35.1.D1	YES	1	37.059		0.003603892
191	35	1	D	35.1.D1	YES	2	35.623		0.006655659
192	35	1	D	35.1.D1	YES	3	34.155		0.01246084
193	35	1	E	35.1.E1	YES	1	35.123		0.008240536
194	35	1	E	35.1.E1	YES	2	36.161		0.005289037
195	35	1	E	35.1.E1	YES	3	28.075		0.167325508
196	35	1	F	35.1.F1	YES	1	36.875		0.003898604
197	35	1	F	35.1.F1	YES	2	NA	NA	
198	35	1	F	35.1.F1	YES	3	36.229		0.005137604
199	35	10	G	35.10.G1	YES	1	32.808		0.022154122
200	35	10	G	35.10.G1	YES	2	32.229		0.028371069
201	35	10	G	35.10.G1	YES	3	32.978		0.02060225
202	35	10	H	35.10.H1	YES	1	37.588		0.002874929
203	35	10	H	35.10.H1	YES	2	NA	NA	
204	35	10	H	35.10.H1	YES	3	35.988		0.005694729
205	35	10	I	35.10.I1	YES	1	33.875		0.014044147
206	35	10	I	35.10.I1	YES	2	34.255		0.011939728
207	35	10	I	35.10.I1	YES	3	34.703		0.009860014
208	35	30	J	35.30.J1	YES	1	29.092		0.108362597

209	35	30	J	35.30.J1	YES	2	29.5		0.091029818
210	35	30	J	35.30.J1	YES	3	29.974		0.074343507
211	35	30	K	35.30.K1	YES	1	29.438		0.093473059
212	35	30	K	35.30.K1	YES	2	29.801		0.080045978
213	35	30	K	35.30.K1	YES	3	29.736		0.08229982
214	35	30	L	35.30.L1	YES	1	27.415		0.221825328
215	35	30	L	35.30.L1	YES	2	28.347		0.148969833
216	35	30	L	35.30.L1	YES	3	27.918		0.178932886
217	42	0.5	A	42.0.5.A1	YES	1	NA	NA	
218	42	0.5	A	42.0.5.A1	YES	2	35.56		0.004795256
219	42	0.5	A	42.0.5.A1	YES	3	NA	NA	
220	42	0.5	B	42.0.5.B1	YES	1	NA	NA	
221	42	0.5	B	42.0.5.B1	YES	2	NA	NA	
222	42	0.5	B	42.0.5.B1	YES	3	NA	NA	
223	42	0.5	C	42.0.5.C1	YES	1	NA	NA	
224	42	0.5	C	42.0.5.C1	YES	2	NA	NA	
225	42	0.5	C	42.0.5.C1	YES	3	NA	NA	
226	42	1	D	42.1.D1	YES	1	32.352		0.018736727
227	42	1	D	42.1.D1	YES	2	37.088		0.002505492
228	42	1	D	42.1.D1	YES	3	NA	NA	
229	42	1	E	42.1.E1	YES	1	NA	NA	
230	42	1	E	42.1.E1	YES	2	33.776		0.010232061
231	42	1	E	42.1.E1	YES	3	31.991		0.021842326
232	42	1	F	42.1.F1	YES	1	NA	NA	
233	42	1	F	42.1.F1	YES	2	32.546		0.01725442
234	42	1	F	42.1.F1	YES	3	NA	NA	
235	42	10	G	42.10.G1	YES	1	NA	NA	
236	42	10	G	42.10.G1	YES	2	NA	NA	
237	42	10	G	42.10.G1	YES	3	NA	NA	
238	42	10	H	42.10.H1	YES	1	NA	NA	
239	42	10	H	42.10.H1	YES	2	32.011		0.021657527
240	42	10	H	42.10.H1	YES	3	NA	NA	
241	42	10	I	42.10.I1	YES	1	NA	NA	
242	42	10	I	42.10.I1	YES	2	NA	NA	
243	42	10	I	42.10.I1	YES	3	36.48		0.00324392
244	42	30	J	42.30.J1	YES	1	33.864		0.009856596
245	42	30	J	42.30.J1	YES	2	NA	NA	
246	42	30	J	42.30.J1	YES	3	NA	NA	
247	42	30	K	42.30.K1	YES	1	33.676		0.010676116
248	42	30	K	42.30.K1	YES	2	NA	NA	
249	42	30	K	42.30.K1	YES	3	NA	NA	
250	42	30	L	42.30.L1	YES	1	33.682		0.010648937
251	42	30	L	42.30.L1	YES	2	31.445		0.027544627
252	42	30	L	42.30.L1	YES	3	31.162		0.031063538
253	0	0.5	A	0.0.5.A1	NO	1	38.006		2.73E-05
254	0	0.5	A	0.0.5.A1	NO	2	31.649		0.002195479
255	0	0.5	A	0.0.5.A1	NO	3	NA	NA	
256	0	0.5	B	0.0.5.B1	NO	1	NA	NA	
257	0	0.5	B	0.0.5.B1	NO	2	NA	NA	
258	0	0.5	B	0.0.5.B1	NO	3	NA	NA	
259	0	0.5	C	0.0.5.C1	NO	1	NA	NA	
260	0	0.5	C	0.0.5.C1	NO	2	32.892		0.000930713
261	0	0.5	C	0.0.5.C1	NO	3	NA	NA	

262	0	1	D	0.1.D1	NO	1	27.274	0.045016665
263	0	1	D	0.1.D1	NO	2	27.298	0.04427687
264	0	1	D	0.1.D1	NO	3	27.143	0.049278068
265	0	1	E	0.1.E1	NO	1	27.355	0.042568217
266	0	1	E	0.1.E1	NO	2	28.969	0.013967782
267	0	1	E	0.1.E1	NO	3	28.676	0.017099504
268	0	1	F	0.1.F1	NO	1	27.536	0.037567526
269	0	1	F	0.1.F1	NO	2	27.734	0.032767422
270	0	1	F	0.1.F1	NO	3	27.491	0.038753046
271	0	10	G	0.10.G1	NO	1	25.939	0.113154847
272	0	10	G	0.10.G1	NO	2	24.641	0.277254473
273	0	10	G	0.10.G1	NO	3	25.319	0.173612188
274	0	10	H	0.10.H1	NO	1	25.735	0.13026945
275	0	10	H	0.10.H1	NO	2	26.65	0.069259565
276	0	10	H	0.10.H1	NO	3	27.814	0.031006609
277	0	10	I	0.10.I1	NO	1	25.373	0.167258542
278	0	10	I	0.10.I1	NO	2	27.403	0.041180598
279	0	10	I	0.10.I1	NO	3	27.5	0.038512986
280	0	30	J	0.30.J1	NO	1	24.663	0.273074948
281	0	30	J	0.30.J1	NO	2	24.534	0.298512498
282	0	30	J	0.30.J1	NO	3	25.226	0.18512551
283	0	30	K	0.30.K1	NO	1	25.162	0.193489174
284	0	30	K	0.30.K1	NO	2	23.962	0.443074506
285	0	30	K	0.30.K1	NO	3	25.296	0.176391138
286	0	30	L	0.30.L1	NO	1	27.39	0.041551883
287	0	30	L	0.30.L1	NO	2	24.62	0.281303679
288	0	30	L	0.30.L1	NO	3	24.621	0.281109526
289	7	0.5	A	7.0.5.A1	NO	1	NA	NA
290	7	0.5	A	7.0.5.A1	NO	2	NA	NA
291	7	0.5	A	7.0.5.A1	NO	3	NA	NA
292	7	0.5	B	7.0.5.B1	NO	1	NA	NA
293	7	0.5	B	7.0.5.B1	NO	2	NA	NA
294	7	0.5	B	7.0.5.B1	NO	3	NA	NA
295	7	0.5	C	7.0.5.C1	NO	1	NA	NA
296	7	0.5	C	7.0.5.C1	NO	2	NA	NA
297	7	0.5	C	7.0.5.C1	NO	3	NA	NA
298	7	1	D	7.1.D1	NO	1	30.902	0.0036772
299	7	1	D	7.1.D1	NO	2	37.071	5.20E-05
300	7	1	D	7.1.D1	NO	3	29.609	0.008978905
301	7	1	E	7.1.E1	NO	1	NA	NA
302	7	1	E	7.1.E1	NO	2	38.863	1.51E-05
303	7	1	E	7.1.E1	NO	3	36.5	7.71E-05
304	7	1	F	7.1.F1	NO	1	20.65	4.360940988
305	7	1	F	7.1.F1	NO	2	NA	NA
306	7	1	F	7.1.F1	NO	3	37.349	4.29E-05
307	7	10	G	7.10.G1	NO	1	31.213	0.002966633
308	7	10	G	7.10.G1	NO	2	31.485	0.002458695
309	7	10	G	7.10.G1	NO	3	31.464	0.002494603
310	7	10	H	7.10.H1	NO	1	29.155	0.012284439
311	7	10	H	7.10.H1	NO	2	30.912	0.003651899
312	7	10	H	7.10.H1	NO	3	NA	NA
313	7	10	I	7.10.I1	NO	1	33.35	0.000678398
314	7	10	I	7.10.I1	NO	2	37.875	2.98E-05

315	7	10	I	7.10.I1	NO	3	34.093	0.000406159
316	7	30	J	7.30.J1	NO	1	37.594	3.62E-05
317	7	30	J	7.30.J1	NO	2	31.78	0.002005621
318	7	30	J	7.30.J1	NO	3	33.704	0.000531297
319	7	30	K	7.30.K1	NO	1	25.417	0.16225381
320	7	30	K	7.30.K1	NO	2	24.82	0.245022179
321	7	30	K	7.30.K1	NO	3	26.825	0.061377048
322	7	30	L	7.30.L1	NO	1	27.816	0.030963823
323	7	30	L	7.30.L1	NO	2	27.896	0.029299929
324	7	30	L	7.30.L1	NO	3	28.258	0.022820273
325	14	0.5	A	14.0.5.A1	NO	1	NA	NA
326	14	0.5	A	14.0.5.A1	NO	2	NA	NA
327	14	0.5	A	14.0.5.A1	NO	3	NA	NA
328	14	0.5	B	14.0.5.B1	NO	1	NA	NA
329	14	0.5	B	14.0.5.B1	NO	2	NA	NA
330	14	0.5	B	14.0.5.B1	NO	3	NA	NA
331	14	0.5	C	14.0.5.C1	NO	1	NA	NA
332	14	0.5	C	14.0.5.C1	NO	2	NA	NA
333	14	0.5	C	14.0.5.C1	NO	3	NA	NA
334	14	1	D	14.1.D1	NO	1	26.935	0.058131921
335	14	1	D	14.1.D1	NO	2	27.542	0.038694825
336	14	1	D	14.1.D1	NO	3	27.181	0.049292189
337	14	1	E	14.1.E1	NO	1	NA	NA
338	14	1	E	14.1.E1	NO	2	NA	NA
339	14	1	E	14.1.E1	NO	3	NA	NA
340	14	1	F	14.1.F1	NO	1	NA	NA
341	14	1	F	14.1.F1	NO	2	NA	NA
342	14	1	F	14.1.F1	NO	3	NA	NA
343	14	10	G	14.10.G1	NO	1	NA	NA
344	14	10	G	14.10.G1	NO	2	34.126	0.000468118
345	14	10	G	14.10.G1	NO	3	37.79	4.01E-05
346	14	10	H	14.10.H1	NO	1	NA	NA
347	14	10	H	14.10.H1	NO	2	37.091	6.41E-05
348	14	10	H	14.10.H1	NO	3	NA	NA
349	14	10	I	14.10.I1	NO	1	36.161	0.000119606
350	14	10	I	14.10.I1	NO	2	NA	NA
351	14	10	I	14.10.I1	NO	3	NA	NA
352	14	30	J	14.30.J1	NO	1	27.741	0.033861249
353	14	30	J	14.30.J1	NO	2	28.763	0.017064393
354	14	30	J	14.30.J1	NO	3	29.643	0.009458667
355	14	30	K	14.30.K1	NO	1	28.226	0.024460665
356	14	30	K	14.30.K1	NO	2	27.758	0.033477458
357	14	30	K	14.30.K1	NO	3	29.618	0.00961856
358	14	30	L	14.30.L1	NO	1	32.469	0.001421945
359	14	30	L	14.30.L1	NO	2	31.045	0.003694534
360	14	30	L	14.30.L1	NO	3	30.585	0.005029378
361	21	0.5	A	21.0.5.A1	NO	1	NA	NA
362	21	0.5	A	21.0.5.A1	NO	2	NA	NA
363	21	0.5	A	21.0.5.A1	NO	3	NA	NA
364	21	0.5	B	21.0.5.B1	NO	1	NA	NA
365	21	0.5	B	21.0.5.B1	NO	2	NA	NA
366	21	0.5	B	21.0.5.B1	NO	3	NA	NA
367	21	0.5	C	21.0.5.C1	NO	1	35.701	0.00016282

368	21	0.5	C	21.0.5.C1	NO	2	NA	NA		
369	21	0.5	C	21.0.5.C1	NO	3	39.233			1.52E-05
370	21	1	D	21.1.D1	NO	1	34.603			0.000339978
371	21	1	D	21.1.D1	NO	2	NA	NA		
372	21	1	D	21.1.D1	NO	3	NA	NA		
373	21	1	E	21.1.E1	NO	1	NA	NA		
374	21	1	E	21.1.E1	NO	2	NA	NA		
375	21	1	E	21.1.E1	NO	3	NA	NA		
376	21	1	F	21.1.F1	NO	1	NA	NA		
377	21	1	F	21.1.F1	NO	2	NA	NA		
378	21	1	F	21.1.F1	NO	3	NA	NA		
379	21	10	G	21.10.G1	NO	1	36.307			0.000108452
380	21	10	G	21.10.G1	NO	2	NA	NA		
381	21	10	G	21.10.G1	NO	3	34.647			0.000330094
382	21	10	H	21.10.H1	NO	1	35.603			0.000173878
383	21	10	H	21.10.H1	NO	2	NA	NA		
384	21	10	H	21.10.H1	NO	3	NA	NA		
385	21	10	I	21.10.I1	NO	1	NA	NA		
386	21	10	I	21.10.I1	NO	2	35.877			0.000144695
387	21	10	I	21.10.I1	NO	3	38.007			3.47E-05
388	21	30	J	21.30.J1	NO	1	38.289			2.87E-05
389	21	30	J	21.30.J1	NO	2	NA	NA		
390	21	30	J	21.30.J1	NO	3	NA	NA		
391	21	30	K	21.30.K1	NO	1	26.464			0.079720958
392	21	30	K	21.30.K1	NO	2	27.08			0.052746041
393	21	30	K	21.30.K1	NO	3	26.578			0.073854179
394	21	30	L	21.30.L1	NO	1	34.693			0.000320068
395	21	30	L	21.30.L1	NO	2	34.272			0.000424462
396	21	30	L	21.30.L1	NO	3	31.83			0.002182546
397	28	0.5	A	28.0.5.A1	NO	1	NA	NA		
398	28	0.5	A	28.0.5.A1	NO	2	NA	NA		
399	28	0.5	A	28.0.5.A1	NO	3	NA	NA		
400	28	0.5	B	28.0.5.B1	NO	1	NA	NA		
401	28	0.5	B	28.0.5.B1	NO	2	NA	NA		
402	28	0.5	B	28.0.5.B1	NO	3	NA	NA		
403	28	0.5	C	28.0.5.C1	NO	1	NA	NA		
404	28	0.5	C	28.0.5.C1	NO	2	NA	NA		
405	28	0.5	C	28.0.5.C1	NO	3	NA	NA		
406	28	1	D	28.1.D1	NO	1	NA	NA		
407	28	1	D	28.1.D1	NO	2	NA	NA		
408	28	1	D	28.1.D1	NO	3	NA	NA		
409	28	1	E	28.1.E1	NO	1	NA	NA		
410	28	1	E	28.1.E1	NO	2	NA	NA		
411	28	1	E	28.1.E1	NO	3	NA	NA		
412	28	1	F	28.1.F1	NO	1	NA	NA		
413	28	1	F	28.1.F1	NO	2	NA	NA		
414	28	1	F	28.1.F1	NO	3	NA	NA		
415	28	10	G	28.10.G1	NO	1	NA	NA		
416	28	10	G	28.10.G1	NO	2	NA	NA		
417	28	10	G	28.10.G1	NO	3	NA	NA		
418	28	10	H	28.10.H1	NO	1	NA	NA		
419	28	10	H	28.10.H1	NO	2	NA	NA		
420	28	10	H	28.10.H1	NO	3	NA	NA		

421	28	10	I	28.10.I1	NO	1	NA	NA	
422	28	10	I	28.10.I1	NO	2	NA	NA	
423	28	10	I	28.10.I1	NO	3	NA	NA	
424	28	30	J	28.30.J1	NO	1	NA	NA	
425	28	30	J	28.30.J1	NO	2	33.615		0.003347131
426	28	30	J	28.30.J1	NO	3	34.45		0.002062772
427	28	30	K	28.30.K1	NO	1	NA	NA	
428	28	30	K	28.30.K1	NO	2	NA	NA	
429	28	30	K	28.30.K1	NO	3	NA	NA	
430	28	30	L	28.30.L1	NO	1	NA	NA	
431	28	30	L	28.30.L1	NO	2	NA	NA	
432	28	30	L	28.30.L1	NO	3	NA	NA	
433	35	0.5	A	35.0.5.A1	NO	1	NA	NA	
434	35	0.5	A	35.0.5.A1	NO	2	NA	NA	
435	35	0.5	A	35.0.5.A1	NO	3	NA	NA	
436	35	0.5	B	35.0.5.B1	NO	1	NA	NA	
437	35	0.5	B	35.0.5.B1	NO	2	NA	NA	
438	35	0.5	B	35.0.5.B1	NO	3	NA	NA	
439	35	0.5	C	35.0.5.C1	NO	1	NA	NA	
440	35	0.5	C	35.0.5.C1	NO	2	NA	NA	
441	35	0.5	C	35.0.5.C1	NO	3	NA	NA	
442	35	1	D	35.1.D1	NO	1	NA	NA	
443	35	1	D	35.1.D1	NO	2	NA	NA	
444	35	1	D	35.1.D1	NO	3	NA	NA	
445	35	1	E	35.1.E1	NO	1	NA	NA	
446	35	1	E	35.1.E1	NO	2	NA	NA	
447	35	1	E	35.1.E1	NO	3	NA	NA	
448	35	1	F	35.1.F1	NO	1	NA	NA	
449	35	1	F	35.1.F1	NO	2	NA	NA	
450	35	1	F	35.1.F1	NO	3	NA	NA	
451	35	10	G	35.10.G1	NO	1	NA	NA	
452	35	10	G	35.10.G1	NO	2	NA	NA	
453	35	10	G	35.10.G1	NO	3	NA	NA	
454	35	10	H	35.10.H1	NO	1	NA	NA	
455	35	10	H	35.10.H1	NO	2	NA	NA	
456	35	10	H	35.10.H1	NO	3	NA	NA	
457	35	10	I	35.10.I1	NO	1	NA	NA	
458	35	10	I	35.10.I1	NO	2	NA	NA	
459	35	10	I	35.10.I1	NO	3	NA	NA	
460	35	30	J	35.30.J1	NO	1	NA	NA	
461	35	30	J	35.30.J1	NO	2	38.731		0.000172447
462	35	30	J	35.30.J1	NO	3	NA	NA	
463	35	30	K	35.30.K1	NO	1	38.826		0.000163207
464	35	30	K	35.30.K1	NO	2	37.777		0.000299804
465	35	30	K	35.30.K1	NO	3	35.732		0.000981052
466	35	30	L	35.30.L1	NO	1	NA	NA	
467	35	30	L	35.30.L1	NO	2	NA	NA	
468	35	30	L	35.30.L1	NO	3	NA	NA	
469	42	0.5	A	42.0.5.A1	NO	1	NA	NA	
470	42	0.5	A	42.0.5.A1	NO	2	NA	NA	
471	42	0.5	A	42.0.5.A1	NO	3	NA	NA	
472	42	0.5	B	42.0.5.B1	NO	1	NA	NA	
473	42	0.5	B	42.0.5.B1	NO	2	NA	NA	

474	42	0.5	B	42.0.5.B1	NO	3	NA	NA
475	42	0.5	C	42.0.5.C1	NO	1	NA	NA
476	42	0.5	C	42.0.5.C1	NO	2	NA	NA
477	42	0.5	C	42.0.5.C1	NO	3	NA	NA
478	42	1	D	42.1.D1	NO	1	NA	NA
479	42	1	D	42.1.D1	NO	2	NA	NA
480	42	1	D	42.1.D1	NO	3	NA	NA
481	42	1	E	42.1.E1	NO	1	NA	NA
482	42	1	E	42.1.E1	NO	2	NA	NA
483	42	1	E	42.1.E1	NO	3	NA	NA
484	42	1	F	42.1.F1	NO	1	NA	NA
485	42	1	F	42.1.F1	NO	2	NA	NA
486	42	1	F	42.1.F1	NO	3	NA	NA
487	42	10	G	42.10.G1	NO	1	NA	NA
488	42	10	G	42.10.G1	NO	2	NA	NA
489	42	10	G	42.10.G1	NO	3	NA	NA
490	42	10	H	42.10.H1	NO	1	NA	NA
491	42	10	H	42.10.H1	NO	2	NA	NA
492	42	10	H	42.10.H1	NO	3	NA	NA
493	42	10	I	42.10.I1	NO	1	NA	NA
494	42	10	I	42.10.I1	NO	2	NA	NA
495	42	10	I	42.10.I1	NO	3	NA	NA
496	42	30	J	42.30.J1	NO	1	NA	NA
497	42	30	J	42.30.J1	NO	2	NA	NA
498	42	30	J	42.30.J1	NO	3	NA	NA
499	42	30	K	42.30.K1	NO	1	NA	NA
500	42	30	K	42.30.K1	NO	2	NA	NA
501	42	30	K	42.30.K1	NO	3	NA	NA
502	42	30	L	42.30.L1	NO	1	NA	NA
503	42	30	L	42.30.L1	NO	2	NA	NA
504	42	30	L	42.30.L1	NO	3	NA	NA
