# Supplementary figures and images for: Benzalkonium Chloride Significantly Improves Environmental DNA Detection from Schistosomiasis Snail Vectors in Freshwater Samples
Source: Trop Med Infect Dis. 2025 Jul 22;10(8):201. doi: 10.3390/tropicalmed10080201 (PMC12390305; doi:10.3390/tropicalmed10080201)

**A**

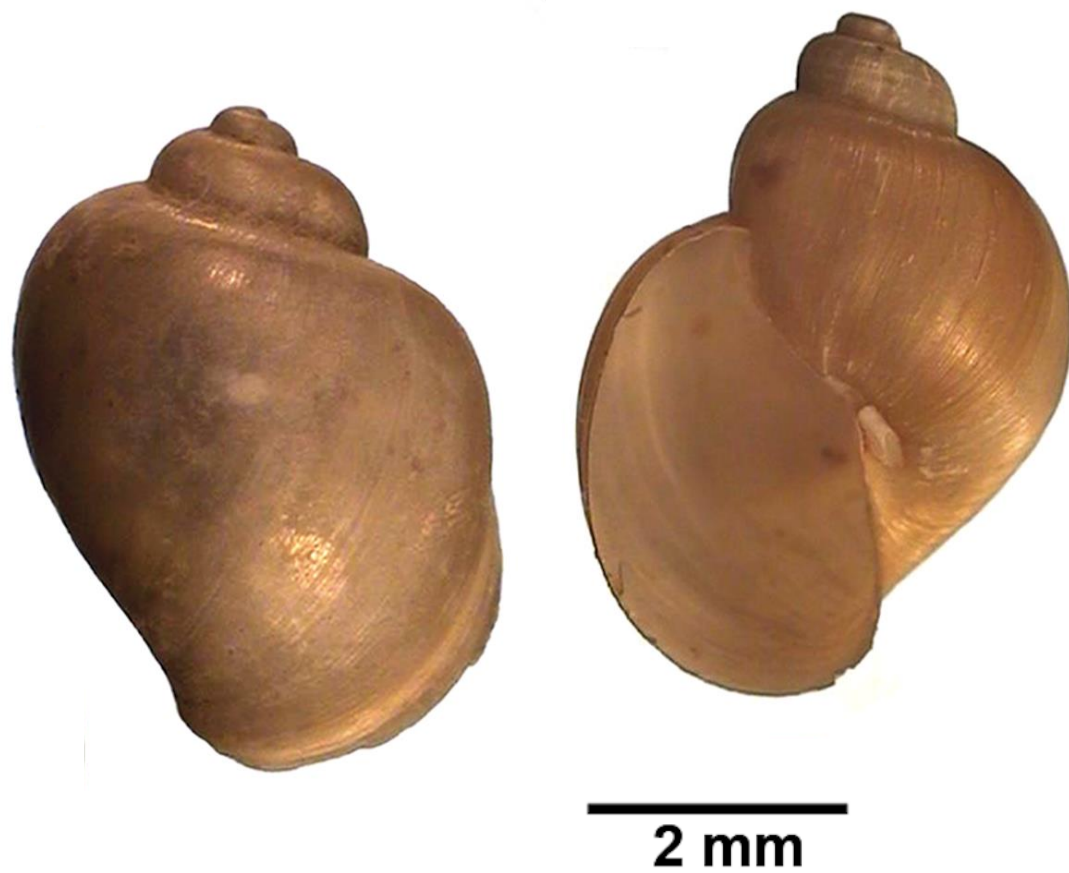

**B**

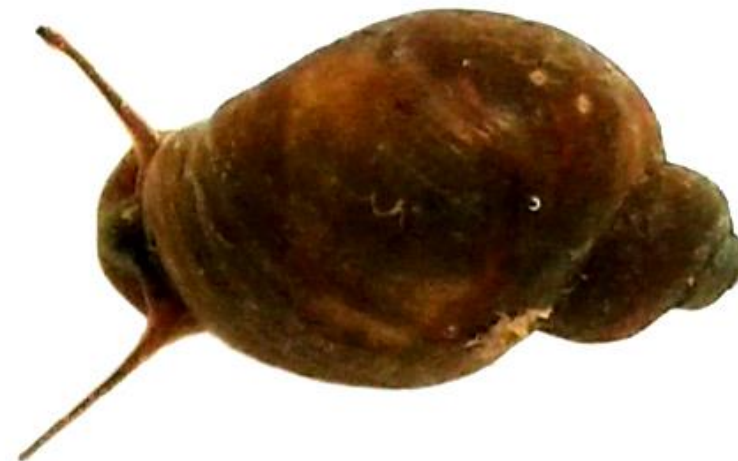

**C**

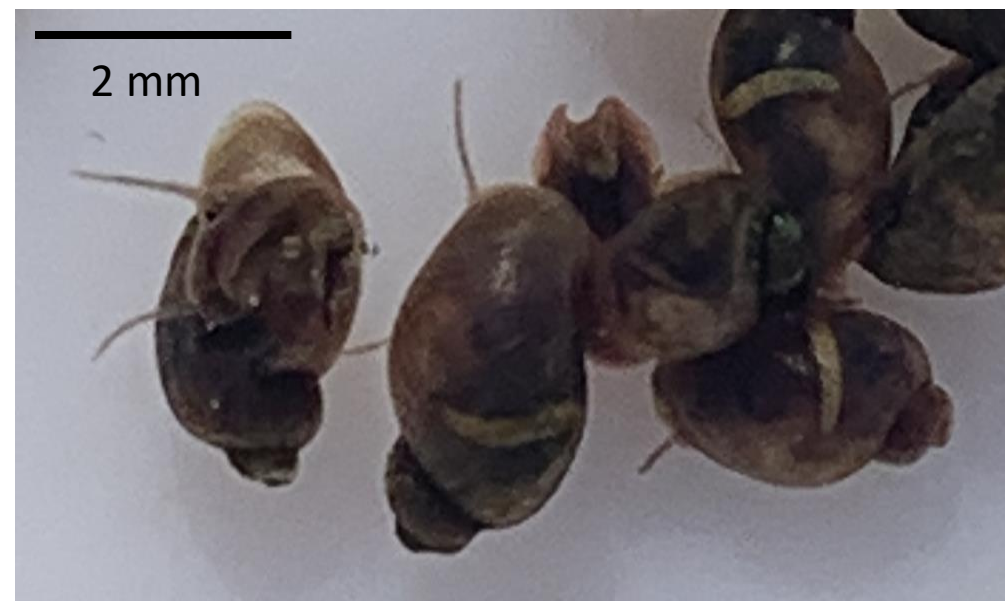

Supplement: Supplementary file 1 [file tropicalmed-10-00201-s001.zip › tropicalmed-3662214 Figure S1.pdf]
